# Current Knowledge in Genetics, Molecular Diagnostic Tools, and Treatments for Mantle Cell Lymphomas

**DOI:** 10.3389/fonc.2021.739441

**Published:** 2021-11-23

**Authors:** Shenon Sethi, Zachary Epstein-Peterson, Anita Kumar, Caleb Ho

**Affiliations:** ^1^ Department of Pathology, Memorial Sloan Kettering Cancer Center, New York, NY, United States; ^2^ Lymphoma Service, Department of Medicine, Memorial Sloan Kettering Cancer Center, New York, NY, United States

**Keywords:** mantle cell lymphoma, genetic, epigenetic, molecular diagnostics, immunochemotherapy, targeted therapy

## Abstract

Mantle Cell lymphoma (MCL) is a mature B-cell lymphoma with a well-known hallmark genetic alteration in most cases, t (11,14)(q13q32)/*CCND1-IGH*. However, our understanding of the genetic and epigenetic alterations in MCL has evolved over the years, and it is now known that translocations involving *CCND2*, or cryptic insertion of enhancer elements of *IGK* or *IGL* gene, can also lead to MCL. On a molecular level, MCL can be broadly classified into two subtypes, conventional MCL (cMCL) and non-nodal MCL (nnMCL), each with different postulated tumor cell origin, clinical presentation and behavior, mutational pattern as well as genomic complexity. This article reviews both the common and rare alterations in MCL on a gene mutational, chromosomal arm, and epigenetic level, in the context of their contribution to the lymphomagenesis and disease evolution in MCL. This article also summarizes the important prognostic factors, molecular diagnostic tools, and treatment options based on the most recent MCL literature.

## Introduction

Mantle cell lymphoma (MCL) is a relatively uncommon subtype of mature B-cell lymphoma with a heterogenous tumor behavior, with most behaving aggressively while others following an indolent clinical course. There have been a lot of advancements in the understanding of the genetics of MCL since the demonstration of t (11,14)(q13;q32)/*CCND1-IGH* as a hallmark feature of MCL in 1990s. The pathogenesis of MCL encompasses complex interactions between the tumor microenvironment, including stromal cells and T-cells, signaling *via* surface immunoglobulins, and tumor cell genetic alterations. Based on the proposed model of molecular pathogenesis, two subtypes of MCL have been recognized, which differ in their clinical and biologic behavior (1) (**Table 1**). The more common conventional MCL (cMCL) arises from expansion of pre-germinal center/naïve-like B cells that are characterized by frequent expression of the transcription factor SOX11, higher likelihood of unmutated immunoglobulin heavy chain variable region (*IGHV*), high genomic complexity, and an aggressive clinical behavior. The less common non-nodal (leukemic) variant (nnMCL), on the other hand, is derived from post-germinal center/memory-like B cells that are generally characterized by mutated *IGHV*, lack of SOX11 expression, low genomic complexity, and an indolent clinical behavior that is partially related to the lack of angiogenic or tumor invasive properties (2–4). The progress in unraveling the pathogenesis and genetic alterations of MCL has also propelled new treatment modalities including molecularly targeted therapies and immunotherapies. This review focuses on the recent developments in the understanding of the mutational-, methylation-, and chromosomal-level alterations seen in MCL and the currently available molecular diagnostic tools and therapy options.

**Table 1:** Two molecular subtypes of mantle cell lymphoma (MCL).

	Conventional MCL (cMCL)	Non-nodal MCL (nnMCL)
**Male: Female**	3–4	1
**Nodal presentation**	82%	38%
**Clinical presentation**	Lymphadenopathy, extranodal	Leukemic, splenomegaly
**Cell-of-origin**	Naïve-like B cell	Memory-like B cell
**Morphology**	Classic/blastoid	Classic/blastoid/plasma cell differentiation
**Immunophenotype**	CD5+ (90–100%), CD200− (90%)	CD5− (25–50%), CD200+ (40–90%)
***IGHV* SHM status**	Unmutated or minimally mutated (IGHV identity >98%)	Hypermutated (IGHV identity <98%)
**SOX11 expression**	Positive	Negative
***ATM* mutation/11q22 deletion**	Common	Rare to Absent
*TP53* mutation/17p13 deletion^*^	Subset of cases	Subset of cases
***CCND1* SHM**	Uncommon	Common
**Genomic complexity/Copy Number Alteration**	Generally High	Generally Low
**Clinical behavior**	Aggressive	Stable/indolent

^*^A statistically significant difference in frequency has not been seen between cMCL and nnMCL.

SHM, Somatic hypermutation.

## Genetics and Pathogenesis

### *CCND1* Translocation and Its Role in Pathogenesis

**Figure 1:**
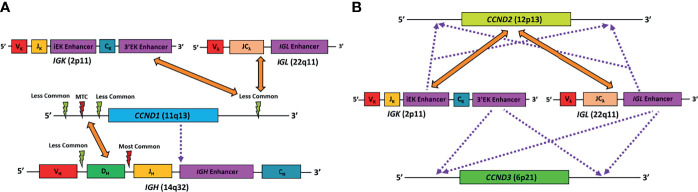
Schematic diagram representing the known major genetic alterations leading to MCL. **(A)** Alterations found in conventional MCL. Orange arrows point to gene translocation partners, red lightning symbol represents major translocation breakpoint, light green lightning symbol represents minor translocation breakpoints, purple dotted line represents cryptic insertion of elements. In *CCND1*-*IGH*, the *IGH* breakpoints are mostly between the *IGHD* and *IGHJ* segments, while others are located between the *IGHV* and *IGHD* segments. The *CCND1* breakpoints are mostly in the region 5’ to the gene, particularly in the major translocation cluster (MTC). In very rare case, *CCND1* coding region can be cryptically inserted into the *IGH* gene, resulting in CCND1 overexpression. Finally, *CCND1* can sometimes pair with *IGK* or *IGL* as translocation partner. In these cases, the *CCND1* breakpoints are more likely to be in the region 3’ to the gene. **(B)** Alterations found in cyclin D1-negative MCL. Orange arrows point to gene translocation partners, and purple dotted line represents cryptic insertion of elements. *CCND2* mostly utilize *IGK* or *IGL*, rather than *IGH*, as translocation partners. On the other hand, conventional translocation of *CCND3* has not been reported in MCL. Alternatively, *IGK* or *IGL* enhancer elements can be cryptically inserted into the vicinity of the *CCND2* or *CCND3* gene, resulting in overexpression of the corresponding gene. VH, VK, Vλ, V gene segments of the *IGH, IGK*, and *IGL* genes, respectively; DH, D gene segment of the *IGH* gene; JH, JK, J gene segments of the *IGH* and *IGK* genes, respectively; JCλ, J and C gene segments of the *IGL* gene.

The translocation t (11,14)(q13;q32) is considered the primary oncogenic event in over 95% of MCL cases that results in the juxtaposition of immunoglobulin heavy-chain (*IGH*) enhancer region on 14q32 next to *CCND1* on 11q13, resulting in its overexpression (1, 5, 6). Irrespective of the molecular subtype, in approximately 90% of the cases, *CCND1* rearrangement (3) occurs in the pro/pre-B cell stage during *IGH* V(D)J recombination and is recombination-activating gene (RAG)-mediated, while in 10% of cases, the translocation occurs in mature B-cell stage during somatic hypermutation (SHM) or class switch recombination and is mediated by B cell-specific activation-induced cytidine deaminase (AID) machinery (3). The mechanism of *CCND1* rearrangment has no apparent clinical or biologic impact. The most common breakpoint on *IGH* locus involves the region between the *IGHD* and *IGHJ* gene segments and occurs during the initial step of *IGH* V(D)J recombination (3, 7). In some cases, the breakpoint occurs during the second step of *IGH* V(D)J recombination involving the region between the *IGHV* and *IGHD* gene segments. The most common breakpoint on 11q32 is located upstream from the *CCND1* gene within the major translocation cluster (MTC) (30% of cases), while in the remainder cases, the breakpoints are located either 5’ or 3’ to the MTC locus (3). Besides having *IGH* as a translocation partner, in a small number of cases, the translocation partner for *CCND1* is the immunoglobulin light chain kappa (*IGK*) or lambda (*IGL*) gene (**Figure 1A**). Furthermore, another small subset of cases does not exhibit *CCND1* translocation (so-called “Cyclin D1-Negative MCL”). Cases with these uncommon alterations will be discussed in the section *Diagnostic Challenges* below.

All types of *CCND1* translocations result in overexpression of *CCND1*, a proto-oncogene that regulates cell cycle transition from G1 to S phase and is often overexpressed or amplified in numerous cancers, including breast, lung, melanoma, and oral squamous cell carcinomas (8). Functions of cyclin D1 include control of cell growth, proliferation, transcription, DNA repair, and migration (9–11). Although Cyclin D1 is not essential for entry into cell cycle progression (12), its amplification/overexpression in human tumors is oncogenic as it allows cancer cells to proliferate independent of extracellular growth signaling cues (8). During lymphomagenesis, cyclin D1 requires cooperation of alterations in other genes, as supported by studies on transgenic mice, which showed that mice solely carrying *CCND1* rearrangement did not develop spontaneous lymphomas (13). The other key alterations that play a role in MCL pathogenesis include deletion of *CDKN2A* locus that encodes p16, a member of the INK4 family of cyclin-dependent kinase inhibitor, amplification of *BMI1* that inhibits *CDKN2A*, deregulation of *TP53 via* mutation or deletion, *MDM2* overexpression, and *ATM* deletion (1, 2, 5).

### Recurrent Driver Mutations and the Main Altered Pathways

Nearly all MCLs carry at least one known driver alteration beside *CCND1* translocation, including gene mutations, copy number alterations (CNAs), and structural variants (SVs) (3). Although cMCL carry a significantly higher number of driver alterations as compared to nnMCL, the overall tumor mutational burden (which is calculated based on the number of point mutations) is similar in the two subtypes. Rather, the differences between the two primarily lie in the number of CNAs and SVs, which in general are higher in cMCL compared to nnMCL (3). Alterations in over 40 driver genes involving eight main pathways have been identified in MCL, namely: DNA damage response, proliferation, cell survival, chromatin remodeling, telomere maintenance, B-cell receptor/Toll-like receptor/NF-kB signaling, NOTCH and RNA regulation (3, 14). The most frequently mutated genes include: *ATM, TP53, CCND1* (SHM or *3’*UTR activation)*, KMT2D, RB1, BIRC3, CDKN2A, CDKN1B, BCOR, NOTCH1*, and *TERT* alterations (promoter mutation, gain/amplification and translocations) (3, 14–17). More details are provided in **Table 2**. Most of the pathogenic mutations seen in MCL patients are somatic in nature. However, more recently, germline mutations in *ATM* and *CHEK2* have also been seen, which raises the possibility that germline mutations in these genes may lead to genetic predisposition to the development of MCL (3, 18). Nonetheless, currently any causal relationship remains speculatory rather than proven.

**Table 2:** Major driver alterations in MCL.

	Genes	Frequency
**DNA damage response**		
	*ATM*	41–50%
	*TP53*	19–28%
	*SAMHD1*	10%
**Cell proliferation and survival**		
	*CCND1* SHM	26%
	*CCND1* 3’UTR activation	21%
	*RB1*	23%
	*CDKN2A*	21%
	*MYC*	15%
	*CDKN1B*	12%
	*SYNE1*	6%
	*DAZAP1*	4%
**Chromatin remodeling**		
	*KMT2D*	14–23%
	*SP140*	13%
	*NSD2*	10-12%
	*SMARCA4*	9%
	*SMARCB1*	4%
**Telomere maintenance**		
	*TERT*	15%
**B-cell receptor/Toll-like receptor/NF-kB signaling**		
	*BCOR*	22%
	*CARD11*	9%
	*BIRC3*	5%
	*TRAF2*	6-22%
**NOTCH regulation**		
	*NOTCH1*	5-14%
	*NOTCH2*	5%
**RNA regulation**		
	*HNRPH1*	6%
**Protein ligase**		
	*UBR5*	6-18%

While mutational profile is not necessarily specific to a particular type of lymphoma, mutations in *CCND1, RB1, CTNNA2, NSD2*, and to a lesser degree, certain types of mutations in *ATM*, are predominantly seen in MCL in comparison to other common mature B- and T-cell lymphomas (14, 15). The presence of these mutations may be a helpful clue in rare diagnostically-challenging cases of MCL.

Alterations of *ATM*, a gene involved in the DNA damage repair pathway, are associated with shorter telomere length in MCL in comparison to MCL with wild-type *ATM*, and consequently, chromosomal instability has been found to be significantly more in MCL with mutated *ATM* (3, 18). Apart from *ATM*, there are likely other genes that also play a role in chromosomal instability, as suggested by the observation that high chromosomal instability can be associated with the blastoid variants of MCL irrespective of the *ATM* gene mutation status. Interestingly, *ATM* mutations are seen mostly in cMCL but not nnMCL, and are commonly truncating mutations or missense mutations involving the PI3K domain (1, 18). In contrast, even though *ATM* mutations are also seen in chronic lymphocytic leukemia (CLL), they are present at a much lower frequency (10–15% in CLL vs 40–75% in MCL) and commonly are missense mutations distributed in different areas of the genes.

Among the other frequent mutations found in MCL, *CCND1* SHM are predominantly seen in nnMCL (3, 14). Alterations in *TP53* have been reported to be either equally distributed among the two subtypes or slightly more enriched in nnMCL, along with *TERT* alterations (3, 14, 19).

### Chromosomal Arm-Level Abnormalities

Overall, MCLs are characterized by frequent chromosomal arm-level abnormalities including gains of chromosomal arm 3q25-29, 7p22/*CARD11*, 8q24/*MYC*, 10p12/*BMI1*, 12q13/*CDK4*, 13q31/*MIR17HG*, and 18q21/*BCL2* and losses of 1p32, 6q/*TNFAIP3*, 9p21/*CDKN2A* and *CDKN2B*, 9q, 11q22/*ATM*, and *BIRC3*, 13q14/*RB1*, and 17p/*TP53* (20). Note that 13q14 loss is also common in CLL and not specific to MCL. Deletions involving 17p and 11q are often associated with *TP53* and *ATM* mutations, respectively (3, 14). The genomic landscape of cMCL is more complex as they carry a significantly higher number of CNAs and SVs than nnMCL. Due to high chromosomal instability, complex alterations such as chromoplexy and chromothripsis are also more frequently observed in cMCL, while breakage–fusion–bridge (BFB) cycles have only been seen in cMCL. The following alterations are exclusively seen in cMCL and not nnMCL: deletions of 1p, 10p, and 19p; and gain of 7p (3). **Table 3** summarizes the chromosomal arm-level abnormalities seen in MCL.

**Table 3:** Major chromosomal-level alterations in MCL.

Chromosomal arm involved	Important genes involved	Frequency
**Gains**		
3q25–q29	*BCL6, TP63*	39%
7p22	*CARD11*	20%
8q24	*MYC*	19%
10p12	*BMI1*	9%
11q13	*CCND1*	9%
12q13–15	*CDK4, STAT6, KMT2D, MDM2*	5%
13q31	*MIR17HG*	8%
18q21	*BCL2*	10%
**Losses**		
1p32	*CDKN2C*	35%
6q	*TNFAIP3*	28%
9p21	*CDKN2A, CDKN2B*	23%
9q22	*CDC14B, FANCC, GAS1*	24%
11q22	*ATM, BIRC3*	34%
13q14	*RB1, SETDB2, DLEU1, DLEU2*	40%
13q33–q34	*CUL4A, ING1, IRS2*	35%
17p13	*TP53*	33%

### Molecular Subtypes of MCL

Distinguishing between the two subtypes of MCL is important prognostically and therapeutically. Among the two molecular subtypes, there are also several genes that are differentially expressed on a mRNA level. *HDGFRP3, FARP1, CSNK1E, SETMAR, HMGB3, LGALS3BP, PON2, CDK2AP1, DBN1, CNR1, CNN3, PLXNB1, DCHS1 NREP, MIML, FNBP1L, FHL1*, and *SOX11* are upregulated in cMCL, while *CD200, BTLA*, and *SLAMF1* are upregulated in nnMCL (19, 21). Based on this differential gene expression pattern, Clot et al. developed a 16-gene assay (L-MCL16 assay) using the NanoString nCounter^®^ platform (NanoString Technologies, Seattle, WA) that can utilize peripheral blood samples to distinguish between the two subtypes of MCL; however, one of the caveats is that it requires ≥60% tumor cell content (19), limiting the utility of this assay on a broader scale. It is also worth noting that a small number of patients in the validation cohort were in the “undetermined” category. As an alternative, a short 3-gene signature comprised of *SOX11, HDGFRP3*, and *DBN1* can also be used reliably to distinguish cMCL from nnMCL in leukemic samples with at least 30% involvement, where low expression profile is associated with the nnMCL subtype (22). The *RNA Gene Expression Profiling* section under *Important Molecular Diagnostic Tools for Genetic Alterations* below provides some important caveats related to gene expression profiling and the nCounter^®^ platform.

### Methylation Profile, Epigenetic Alterations, and SOX11 Expression

Besides looking at the gene expression profile and gene mutation pattern, the DNA methylation study represents another methodology that can shed insights on the tumor biology. Queiros et al. compared the DNA methylation profile of MCL cases and normal non-neoplastic B cells at different stages of maturation using principal component analysis of the first two main components. In the first principal component (PC), all MCL cases have been found to be more similar to germinal center-experienced B-cells, suggesting that MCL originates from cells with some degree of antigenic experience. However, the second PC showed that within MCL, the cases can be divided into two clusters that are biologically and clinically distinct. The first cluster represents mostly the cMCL, and as expected, has a pattern more resembling the germinal center-inexperienced cells, showing either the absence of or low but variable level of *IGHV* SHM. The second cluster represents most cases of nnMCL, and also as expected, has pattern more resembling the germinal center-experienced cells (3, 23).

One of the main distinguishing factors between cMCL and nnMCL is expression of SOX11, which is usually higher in the former and lower to absent in the latter. SOX11 expression alone is not reliable for classification, because there is a spectrum of SOX11 expression among cases in each subtype, and a subset of cMCL and nnMCL cases can have overlapping level of SOX11 expression (19). The mechanism of SOX11 expression is thought not to be due to gene mutation, but rather hypomethylation of a distant enhancer region of *SOX11*, leading to alteration of the 3-dimensional chromatin pattern with eventual activation of transcription of *SOX11*. This methylation change has been observed in cMCL, but is not seen in normal B-cells or most nnMCL (23). The precise role of SOX11 in MCL pathogenesis is still being explored. Mouse models overexpressing SOX11 have shown oligoclonal expansions of CD5+/CD23− B-cells, similar to MCL (24). SOX11 represses BCL6 expression, blocking the entry of B-cells into germinal center and thus may be integral for determining the cell of origin for MCL subtypes (25). Additionally, SOX11 promotes PAX5 expression, which in turn blocks plasmacytic differentiation, locking the cell in the mature B-cell stage, and has been previously implicated as an oncogenic mechanism in other B-cell lymphomas (26–28).

### Tumor Microenvironment

The role of tumor microenvironment in lymphomagenesis and drug resistance has been previously demonstrated in several B-cell malignancies. Recent studies show that the stromal interactions in MCL *via* adhesion molecules and cytokines influence activation of multiple pathways including B-cell receptor (BCR) and NF-kB, promoting cell proliferation and survival as well as trafficking to tumor supportive tissue microenvironments. When compared to peripheral blood, it has been observed that the lymph node microenvironment in MCL fosters BCR and NF-kB signaling (29). In the bone marrow, another common site of MCL involvement, stromal cells upregulate expression of focal adhesion kinase (FAK), CXCR4 and CXCR5 chemokine receptors, and VLA-4 adhesion molecules, likely resulting in downstream activation of the NF-kB and PI3K/AKT pathways (30, 31). SOX11 also interacts with the microenvironment *via* the FAK/PI3K/AKT pathway axis to promote cell growth, angiogenesis and cell migration (25, 26, 32).

When active, these pathways may serve as potential targets for inhibition, offering alternate treatment strategies from conventional chemotherapy agents. Discussion of all these targets is beyond the scope of this review article, but one class of therapy, small molecular inhibitor of Bruton tyrosine kinase (BTK), an essential component for BCR signaling, has gained a lot of attention in recent years as a treatment for various B-cell lymphomas. One irreversible inhibitor in this class, ibrutinib, has shown efficacy in MCL, as well as in CLL and activated B-cell subtype of diffuse large B-cell lymphoma (33–37). Nevertheless, a subset of patient showed either intrinsic resistance or developed acquired resistance to ibrutinib. Other approved BTK inhibitors include zanubrutinib and acalabrutinib. Newer generation of BTK inhibitors are under development, and may potentially overcome the therapy resistance observed with ibrutinib. These were will be discussed more in the *Current Treatment Options* section below.

## Diagnostic Challenges

### Cryptic *CCND1* Rearrangements

At most institutions, the diagnosis of MCL relies on demonstrating the classic histopathologic features and tumor cell immunophenotype (CD5+, cyclin D1+, CD23−, SOX11+/−), but many also perform *IGH*/*CCND1* dual-color dual-fusion fluorescence *in situ* hybridization (FISH) and/or *CCND1* break-apart probe studies to confirm the diagnosis. However, it is important to keep in mind that very rare cases of MCL with cyclin D1 overexpression detectable at the protein level, may have cryptic *CCND1* insertional event into the *IGH* locus that escapes detection by conventional FISH probes or karyotype analysis (38) (**Figure 1A**). These cryptic rearrangements may be more easily detected by whole genome sequencing (WGS) instead.

### *CCND1* Translocations With Non-*IGH* Partners

**Figure 2:**
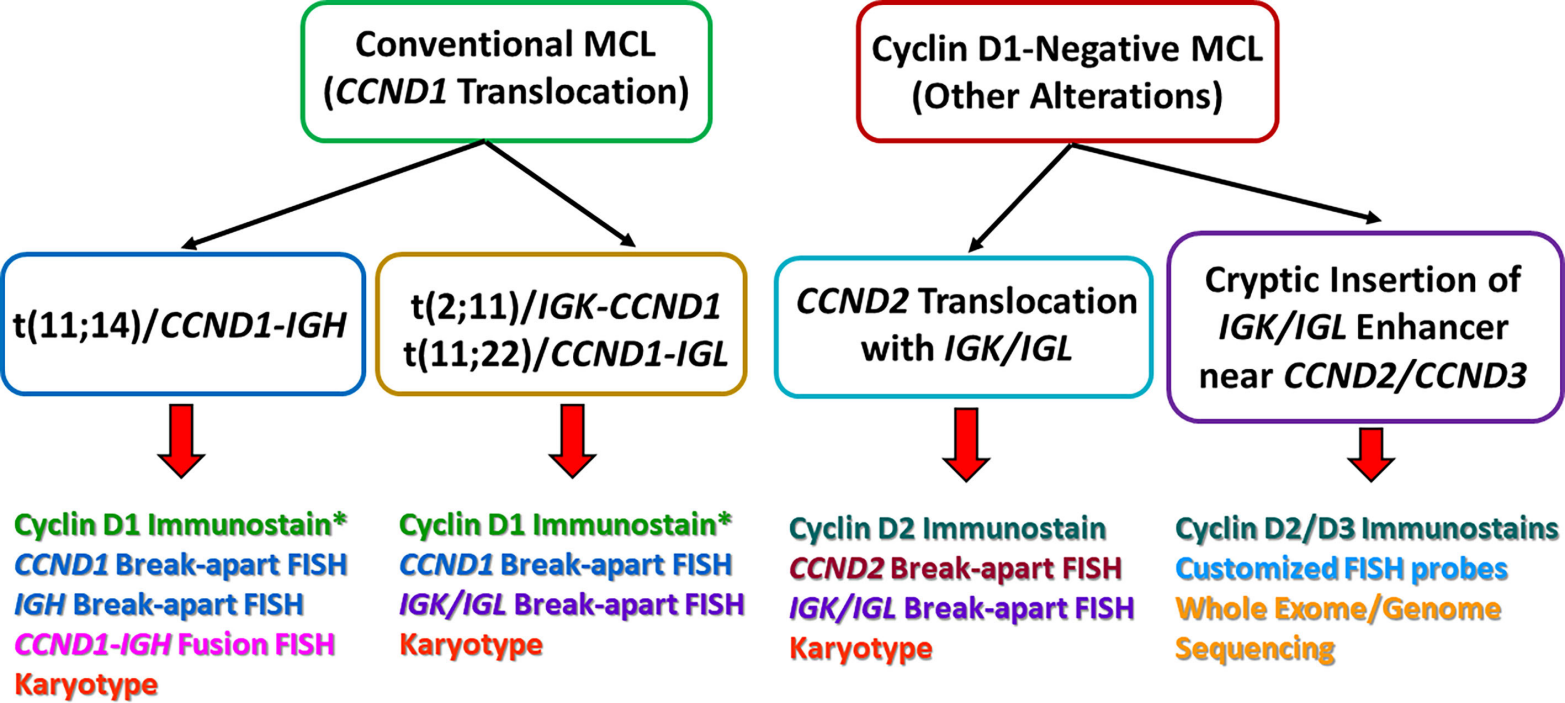
A comparison of the major genetic alterations between conventional mantle cell lymphoma (MCL) and cyclin D1-negative MCL. The bottom of the picture also listed some of the common methods for detection. *In rare cases, MCL can harbor the *CCND1-IGH* translocation, but cyclin D1 immunostain can be negative. This can be due to poor viability of tumor cells, technical issues, as well as mutations within the *CCND1* gene that alter the immunostain antibody epitope.

There are rare reports of MCL with translocations involving *CCND1* and *IGK* (chromosome 2) or *IGL* (chromosome 22) instead of *IGH* (chromosome 14), analogous to *IGK/MYC* or *IGL/MYC* translocations seen in Burkitt lymphoma (39–43) (**Figure 1A**). A case of MCL with t(11, 12) (q13;p11.2) has also been reported (44). The 11q32 breakpoints for these variant translocations are mostly located in the 3’ region, which contrasts with the breakpoints associated with conventional *IGH/CCND1* translocation that are centromeric (5’) from the *CCND1* gene (39, 43). These cases would lack the expected fusion signal pattern by *IGH/CCND1* dual-color dual-fusion FISH, but a helpful clue in such cases would be the presence of an extra signal for *CCND1* as a consequence of one intact and one split *CCND1* signal. Use of *CCND1* break-apart probe, *IGK/IGL* break-apart probes or *IGK/CCND1* or *IGL/CCND1* dual-color dual-fusion probes could help prove the presence of these variant translocations, and aid in the diagnosis of MCL (**Figure 2**). While very rare, instead of conventional gene translocation involving *CCND1*, cryptic insertion of *IGK* and *IGL* enhancers in the vicinity of *CCND1* can occur, which also leads to cyclin D1 overexpression and MCL phenotype, and would be missed by *CCND1* break-apart probe (45). Such cases may be detectable only with *IGK/IGL* break-apart probes, *IGK/CCND1* or *IGL/CCND1* dual-color dual-fusion probes, or WGS assays.

### Non-*CCND1* Translocation/Cyclin D1-Negative MCL

A minor subgroup of MCL lacks *CCND1* translocation as well as cyclin D1 expression (so-called “cyclin D1-negative MCL”). This subgroup has otherwise similar morphologic, phenotypic (including expression of *SOX11*) and genomic profiles as the cyclin D1-positive MCL (46–49). Nearly all of these cases show overexpression of either cyclin D2 or cyclin D3. Immunohistochemistry for cyclin D2 or cyclin D3 may serve as a screening tool for such cases. The major alteration in these cases involves the translocation of *CCND2* (55–70% of cyclin D1-negative MCL) with an immunoglobulin gene, preferably with *IGK* and *IGL* rather than *IGH*. Most of the *CCND2* translocations can be detected by break-apart probes (*CCND2* or *IGK/IGL*). In the remaining cases, the lymphomas harbor cryptic insertion of enhancer elements of *IGK* or *IGL* into the vicinity of the *CCND2 or CCND3* genes (**Figure 1B**). Due to the small size of the inserted enhancer element, such cases will be missed by conventional break-apart probes and require the use of either special customized *IGK* enhancer-*CCND2/CCND3* fusion probes or WGS for detection (46, 47) (**Figure 2**). Older studies describing cyclin D1-negative MCL, confirmed by gene expression signature, and demonstrating cyclin D2 or D3 overexpression but lacking demonstrable rearrangement by conventional break-apart probes, possibly represented such cases (49).

Even more rare are cyclin D1-negative cases (<10%) lacking protein expression of cyclin D1, cyclin D2 and cyclin D3. Such cases show concomitant upregulation of *CCNE1* and *CCNE2*, but lack a demonstrable relevant structural rearrangement (46). These cases show high genomic complexity and are associated with blastoid morphology (46).

Immunohistochemical (IHC) stains for SOX11, cyclin D2 and cyclin D3 may be helpful as part of the secondary panel in cyclin D1-negative lymphoid malignancies that have other features suggestive of MCL. Unlike cyclin D1, neither SOX11, cyclin D2 nor cyclin D3 expression is specific for MCL. Cyclin D2 or D3 expression can also be seen in CLL, follicular lymphoma or splenic marginal zone lymphoma. SOX11 expression has been reported in T-cell and B-cell lymphoblastic lymphoma/leukemia, Burkitt lymphoma, T-cell prolymphocytic leukemia, and rarely in classic Hodgkin lymphoma. Nevertheless, as compared to other B-cell lymphomas, with the exception for some cases of Burkitt lymphomas, the SOX11 mRNA levels in MCL is much higher (48).

### Cyclin D1-Negative Immunostaining Despite t (11; 14)

Finally, an uncommon but potential pitfall in the diagnosis of MCL is the lack of cyclin D1 positivity by IHC stain despite the presence of t (11,14) detected by genetic studies, along with high CCND1 mRNA expression and SOX11 expression. Some potential explanations include mutations in *CCND1* that significantly alter the 3D protein structure of the IHC antibody-binding epitope, suboptimal staining due to technical issues with instrumentation or IHC antibody, and pre-analytical factors such as poor formalin fixation, or low tumor cell viability. One of the most commonly-used commercially-available cyclin D1 IHC antibodies (clone SP4) is a monoclonal antibody that binds to the C-terminus portion of cyclin D1. Mutations that alter the 3’ end of *CCND1*, such as *CCND1* p.D292P, can alter the C-terminus portion of the cyclin D1 protein, and can impair binding of this antibody, resulting in a false negative IHC result (50). In such cases, the use of alternative antibodies that bind to the N-terminus of cyclin D1 will circumvent the problem.

Another mechanism for cyclin D1 IHC false negativity is related to mutations that can affect selected isoforms of *CCND1*. Alternative splicing of *CCND1* produces two major isoforms, the long isoform (cyclin D1a) and the short isoform (cyclin D1b). The short isoform derives from a splicing event that skips exon 5 and includes part of intron 4. Consequentially, cyclin D1b lacks the epitope for the C-terminal-binding cyclin D1 antibody. A defect in the expression of cyclin D1a isoform, which has been seen in association with the *CCND1* p.L6P mutation, has been found to be another reason for cyclin D1 immunostain false negativity, due to the presence of predominantly cyclin D1b isoform that lacks the appropriate antibody epitope (50).

## Prognostic Factors

### Histologic Subtypes of MCL

For practical purposes, the morphologic spectrum of MCL can be broadly divided into the classic and aggressive histologic variants. MCLs with blastoid and pleomorphic morphology are considered aggressive, as they exhibit inferior survival and response to chemotherapy (51, 52). These aggressive variants may arise *de novo* or from progression of an underlying classic variant of MCL, and are associated with high degree of aneuploidy and mutation burden, including *KMT2D* and *KMT2B* mutations (53). Jain et al. performed whole-exome sequencing (WES) in 183 patients of MCL with aggressive histology and found that mutations in *NOTCH2, NOTCH3*, and *UBR5* were exclusive to the blastoid and pleomorphic variants. Additionally, they reported that aggressive histology MCL with Ki-67 proliferation index ≥50% have exclusive mutations in *CCND1, NOTCH1, TP53, SPEN, SMARCA4, RANBP2, KMT2C, NOTCH2, NOTCH3*, and *NSD2* (53). It is also worth noting that since treatments for lymphomas can alter the tumor cell morphology, morphologic features may not be as reliable in predicting aggressive behavior and genomic complexity in the setting of post-treatment relapsed/refractory (R/R) MCL.

### Molecular Subtypes and Genomic Complexity

As mentioned previously, the two molecular subtypes of MCL, cMCL and nnMCL differ in *IGHV* SHM status, gene expression profile and genomic complexity, and are usually associated with aggressive and indolent behavior, respectively (3, 54, 55). Time to treatment and overall survival (OS) from the time of diagnosis is significantly longer for nnMCL patients than cMCL (19, 22). The literature showed that a 5-year OS for cMCL ranged from 32 to 40%, and for nnMCL ranged from 59 to75% (22, 54). Nevertheless, nnMCL may acquire additional genetic alterations and undergo transformation to aggressive variants that confer worse prognoses (see *Secondary Genetic Events* below).

Although the total number of mutated driver genes does not have an impact on prognosis, chromosomal instability in the form of BFB and chromothripsis or high number of CNA and SV are associated with a shorter OS in MCL[3]. The number of CNA (>7), or presence of BFB shows independent prognostic value in multivariate analysis (3). This is also in keeping with the observation that the more aggressive blastoid variant of MCL has higher number of copy number gains and losses (56) (see *Histologic Subtypes of MCL* above).

### Proliferative Activity and DNA Methylation Burden

Immunohistochemical evaluation of Ki-67 proliferative index is part of routine evaluation of MCL as per the European Mantle Cell Lymphoma Pathology Panel, because it is a strong prognostic factor for OS and progression-free survival (PFS) independent of the Mantle Cell Lymphoma International Prognostic Index (MIPI). Consequently, a combination of Ki-67 and MIPI (biologic MIPI) provides a better risk stratification into four groups with significantly different prognoses (51). A Ki-67 >30% is the currently accepted cut-off for high-risk behavior (57).

Tumor cell proliferation can also be assessed on a gene expression level. A gene signature comprised of 20 genes that has been identified as a strong predictor of survival. It is highly expressed in proliferating cells (such as *CDC2, ASPM*, tubulin α, etc.) and correlates with mitotic index, further validating the role of proliferation rate in determining the clinical course in MCL (58). A similar proliferation assay (MCL35), developed by Scott et al., utilizes the NanoString nCounter^®^ platform (NanoString Technologies, Seattle, WA) to assess expression of 17 genes on MCL formalin-fixed paraffin-embedded (FFPE) tissue (expression of 18 other housekeeping genes was also assessed for normalization purposes, for a total of 35 genes assessed). This assay classifies patients treated with R-CHOP (rituximab, cyclophosphamide, doxorubicin, vincristine, and prednisone) into the high-risk, standard-risk, and low-risk groups, and the stratification was thought to have independent prognostication value from the MIPI score (59). The RNA *Gene Expression Profiling* section under *Important Molecular Diagnostic Tools for Genetic Alterations* below provides some important caveats related to gene expression profiling and the nCounter^®^ platform.

Finally, the strength of a proliferation signature, which is predictive of inferior survival in MCL, correlates with the strength of the BCR signaling as well (29). The highly proliferative MCLs show high levels of cyclin D1 mRNA, with the short, truncated variant of cyclin D1a isoform preferentially expressed. This short isoform has a longer half-life and consequently prolongs the oncogenic effect of cyclin D1 (60). The proliferative activity also determines the DNA methylation burden, which has been found to be an independent prognostic factor in MCL. High DNA methylation burden has stronger prognostic value than that of *IGHV* mutation rate and patient’s age, and is associated with a worse clinical outcome (3, 23).

### Secondary Genetic Events

Even in the generally indolent subtype of MCL, nnMCL, the tumors may acquire additional genetic alterations, such as *TP53* mutations and 17p deletions, that may impart an aggressive behavior (22, 54). *TP53* alterations have been shown to be an independent adverse risk factor in MCL irrespective of high Ki-67, high MIPI score or blastoid morphology (14, 61). Other known chromosomal- and mutational-level alterations associated with shorter OS include: loss of *CDKN2A*, loss of *RB1*, loss of 13q33-q34, loss of 9q22-q31, rearrangement of *MYC*, gain of 18q21-q22, and *SP140* mutations (3). In a recent study, *MYC* gene rearrangements, but not extra copies of *MYC*, were found to have a negative impact on OS in multivariate analysis (62).

From a therapeutic target standpoint, some of the secondary genetic alterations involving the BCR signaling, PIK3-AKT, canonical and non-canonical NF-kB pathways such as *NSD2, NOTCH2, UBR5, BIRC3, TRAF2, MAP2K14, KMT2D, CARD11, SMARCA4*, and *BTK*, have been found to be associated with ibrutinib resistance (63). These alterations pose therapeutic challenges, and their detection can help identify patients in need of alternative treatment regimens.

On the other hand, the prognostic role of other secondary genetic events are either more controversial or less established due to limited data available. For example, some studies have reported a negative impact of *NOTCH1* mutations in univariate analysis (14, 64) and in a multivariate Cox regression model that also included IPI and histology (16), while in others, *NOTCH1* mutations did not carry an independent prognostic value due to co-occurrence with *TP53* mutations (61). *NOTCH2* mutations occur independently of *NOTCH1* mutations, and have been found to have significantly lower 3-year OS than the non-mutated cases (0 vs 62%, P = 0.0002 in *NOTCH2*-mutated and wild-type cases, respectively). In one prospective randomized control study of young patients, *KMT2D* mutations were found to be an independent prognostic marker of OS and PFS despite intensive immunochemotherapy and autologous stem cell transplant (61).

Most of the existing literature thus far have focused on the role of *TP53* alterations in MCL. In current clinical practice, in the context of genetic alterations, only *TP53* abnormalities influence treatment decisions (see *Frontline Treatment for MCL* section under *Current Treatment Options* below). Also, a major limitation in many of the existing studies is that it remains unclear if subclonal/low-level alterations in the genes have the same prognostic impact as having the alterations in the entire disease clone. The therapeutic implications of gene alterations other than *TP53* certainly deserve further and more thorough evaluations.

### Variant Translocations/Cryptic Enhancer Insertions

Overall, due to rarity of cases and treatment heterogeneity of MCL with variant translocations or cryptic enhancer insertions reported in the literature, any conclusion regarding their prognoses should be interpreted with caution. The few reported cases of MCL with variant *CCND1* translocations involving *IGK* or *IGL* instead of *IGH* mostly presented as nnMCL and followed a relatively indolent clinical course (39–43). However, no statistical difference in OS has been found among *CCND1* translocated and non-*CCND1* translocated cases. Furthermore, cases with *CCND2* translocations or cryptic insertions of *IGK/IGL* enhancer elements near *CCND2/CCND3* are associated with a similar OS as the *CCND1*-translocated cases (46).

### Measurable/Minimal Residual Disease

Although not part of current routine management strategies, laboratory techniques for assessing MRD may serve as alternatives to 18 fluorodeoxyglucose positron emission tomography/computed tomography (18FDG PET-CT) for lymphoma response assessment and clinical decision making. In the context of MCL, measurement of molecular MRD is usually performed on peripheral blood or bone marrow, using methodology such as high sensitivity flow cytometry, allele-specific oligonucleotide polymerase chain reaction (PCR) or next-generation sequencing (NGS)-based *IGH* clonal rearrangement assay. Each of these can serve as a highly sensitive tool for monitoring tumor response to therapy at all time points during induction and consolidation. These diagnostic tools have been particularly helpful in the design of clinical trials to assess the efficacy of existing and novel drug regimens, and aid in tailoring the intensity and combinations of various frontline treatments. MRD assessment by molecular assays on peripheral blood or bone marrow samples, following initial immunochemotherapy, has been shown to be a strong independent prognostic factor and predictor of sustained clinical response and progression-free survival. However, MRD in MCL is still an emerging concept and prospective randomized clinical trials (such as NCT03267433) are warranted to design and evaluate MRD-guided treatment strategies (65–68). Further details on molecular MRD assays can be found in the section *IGHV Somatic Hypermutation (SHM) Assessment and NGS-Based MRD Assay* below.

## Important Molecular Diagnostic Tools for Genetic Alterations

### Overview

Based on the most recent National Comprehensive Cancer Network (NCCN) guidelines (v.5.2021), besides relevant cell marker assessment by flow cytometry/IHC and cytogenetics karyotype/FISH studies for confirming a diagnosis of MCL, the only molecular testing considered essential is *TP53* gene sequencing, for patients with expected aggressive clinical course (69) (see *Frontline Treatment for MCL* section under *Current Treatment Options* below). *IGHV* sequencing for determination of SHM status is considered helpful under certain circumstances, particularly for determination of clinically indolent MCL. Otherwise, additional molecular studies are considered optional. Other studies such as RNA gene expression and DNA methylation profiling are not performed routinely for clinical purposes, but can aid in the understanding of tumor pathogenesis.

### Mutational Profiling

Detection of mutations in tumor cells can be performed by a variety of methodology, such as allele-specific PCR for detection of specific known mutations, Sanger sequencing for detection of mutation within a specific region, and NGS assays (also known as massive parallel sequencing). Different sequencing platforms and assay kits are available commercially, and discussion of each will be beyond the scope of this article. However, for the purpose of detection of *TP53* mutations, which are distributed throughout the gene rather than being confined to a few codons, Sanger sequencing and NGS assays are usually utilized, rather than allele-specific PCR. Sanger sequencing can be helpful for confirmation of findings, but in general it has worse technical detection sensitivity, is more costly, and inefficient in processing a large number of samples, as compared to the widely-used NGS assays (70).

For NGS assays, the sequencing strategy can be broadly classified into: 1. Targeted sequencing—enrichment of specific genes/exons by amplicon (PCR-based) or hybrid-capture techniques; 2. WES—enrichment of all exonic and surrounding region; 3. WGS—covering the entire genome, including the noncoding and intronic regions. In general, WGS yields the greatest breadth of information, is best suited for detecting larger gene insertions/deletions and SVs, and less prone to certain technical artifacts seen in targeted sequencing and WES. However, it is currently the most costly and time-consuming method for analyses, and due to lower overall coverage, may miss variants in samples with relatively low tumor content, or subclonal, low-level variants (70). Targeted sequencing has been favored in some clinical laboratories, due to the ability to detect variants at lower allele frequencies, lower cost, and the shorter amount of time needed for analyses, which has translated to faster report generation and sample turnaround time. If a targeted sequencing assay is chosen, it should be confirmed that important driver genes in MCL are covered by the assay (See **Table 2**).

Regardless of the breadth of sequencing regions, mutational profiling could be performed by sequencing of the tumor sample only, or paired sequencing of a normal control with the tumor sample. Analysis of a patient’s paired normal and tumor sample allows most germline variants to be identified and filtered out during analyses (71). The main advantage of this approach is that only somatic variants, which are more likely to be pathogenic, are eventually included in the clinical reports. In addition, separate analysis can be performed on the sequencing data from the normal sample for detection of clinically significant germline/inherited variants (72). For paired tumor-normal sequencing of MCL, since there is a possibility of circulating tumor cells, caution should be made if blood sample is used as normal control, which is commonly used in the sequencing of non-hematologic neoplasms. Rather, alternative normal control, such as saliva or buccal swab, should be considered instead.

Finally, it is worth pointing out that the terms “mutation” and “variant” should not be used interchangeably. “Variant” is a broader term that describes nucleotide changes as compared to the reference sequence, and does not imply pathogenicity of the change. The American College of Medical Genetics and Genomics (ACMG) and the Association for Molecular Pathology (AMP) advocated for a 5-tier classification of variants, which include: pathogenic, likely pathogenic, uncertain significance, likely benign, or benign (73). The classification of variants is particularly important for tumor-only sequencing assays, in which germline benign variants (some of which are also known as polymorphisms) can be detected. In the absence of paired tumor-normal sequencing, to help distinguish between somatic and germline benign variants, the variant allele frequencies can be used in conjunction with information from publicly-available databases of somatic variants (e.g., COSMIC: https://cancer.sanger.ac.uk/cosmic), and several population-based databases of germline variants (e.g., dbSNP: https://www.ncbi.nlm.nih.gov/snp; gnomAD: https://gnomad.broadinstitute.org; The International Genome Sample Resource/The 1000 Genomes Project: https://www.internationalgenome.org). As a general rule, due to the clinical implication of assigning a variant as pathogenic, it is recommended to do so only when there is a reasonably high level of confidence in it being so (e.g., listing in multiple reliable databases of known pathogenic variants, available literature on functional studies on that particular variant).

### IGHV Somatic Hypermutation Assessment and NGS-Based MRD Assay

Historically, much of the knowledge of *IGHV* SHM assessment arose from the data for CLL, where the *IGHV* SHM status has served as a key prognostic marker for the past two decades (74). In CLL as well as MCL, the *IGHV* SHM status also appears to be stable over time, regardless of therapy, and has traditionally been assessed by Sanger sequencing of the *IGH* gene VDJ segments. As clinical laboratories increasingly incorporate NGS-based assays into their workflow, and the corresponding sequencing instruments in their laboratories, *IGHV* sequencing by NGS method has also gained in popularity as a replacement for Sanger sequencing.

In terms of *IGHV* sequencing by NGS method, several commercial assays are available, and the same assay is often used for *IGH* clonal rearrangement (B-cell clonality) detection, as well as MRD detection of the known *IGH* disease clone in a post-treatment or follow-up sample. Different assays employ different strategies, but the basic principles remain the same: the use of PCR primer sets to flank and amplify the *IGH* gene VDJ segments [usually the forward primer set is either upstream to or within the framework 1, 2, or 3 (FR1, FR2 or FR3) region of the V segment, or the D segment, while the reverse primer set is in the J gene segment] (75). Some assays may employ multiple primer sets to different regions of the *IGH* gene to overcome the problem of poor primer annealing that can result from high-level somatic mutations within the *IGH* gene. After sequencing, the disease-associated clonal sequence is identified and compared to a reference database, to determine the % of SHM as compared to the germline sequence. The patient- and disease-specific clonal sequence can also be saved, and searched for at very low level in subsequent samples, which form the basis of MRD detection. Besides commercial assays, in recent years, the European Research Initiative on CLL (ERIC) and the EuroClonality-NGS Working Group have collaborated to develop the appropriate primer sets and analysis pipeline for clonal sequence characterization and SHM determination from a NGS-based *IGHV* sequencing assay (74).

### RNA Gene Expression Profiling

Somewhat analogous to the different DNA sequencing strategies mentioned above, global RNA gene expression can be performed to provide a global picture of the different alterations as compared to normal control/non-neoplastic tissue, but is mostly done in research settings. Targeted RNA gene expression profile is more feasible in the clinical setting, although in the case of MCL, it is not currently widely used for routine cases. This may be partially related to the need for RNA extraction from FFPE or blood, which is not performed at some smaller laboratories. For testing on archival FFPE samples, RNA is also not as well-preserved over time, compared to DNA.

Both MCL35 (59) and L-MCL16 (19) assays mentioned previously were designed using the NanoString nCounter^®^ platform and require specific instrumentation from the manufacturer. This is in contrast to the sequencing instrumentation for NGS-based assays, where the same instruments can potentially be adapted for a variety of assays and serve multiple purposes (e.g., targeted mutation profiling assays using reagents/kits from different manufacturers, as well as *IGHV* sequencing by NGS method).

### Single Nucleotide Polymorphism (SNP) Array

SNP array as a method of assessing CNAs has several advantages over conventional karyotype. First of all, while conventional karyotype requires fresh cell cultures, SNP array is a DNA-based assay, and therefore can be performed on existing extracted DNA, or on DNA extracted from FFPE tissue. With routine, conventional G-band karyotyping, chromosomal change >10 Mb can be detected; on the other hand, with newer SNP array assays, the average inter-marker distance is 680 bp, allowing a much higher resolution in detecting small areas of chromosomal-level gain/loss (76). Furthermore, SNP array can detect copy neutral-loss of heterozygosity (CN-LOH), as well as provide information on the level of copy number gain/loss, which are not possible by karyotyping. Nevertheless, unlike karyotype, SNP array cannot detect balanced translocation with no net loss of chromosomal material, and certainly not the partners involved in a translocation. Compared to FISH studies, which are usually much more targeted, and each set of probes assess alterations in a single chromosome, gene, or specific translocation, SNP array has the advantage of providing a broad overview of CNAs across all the chromosomes. On the flip side, since FISH studies assess for alterations in individual cells, in the context of fresh tissue studies, FISH studies can detect abnormalities at a much lower level than SNP array. This can be a consideration for samples with low tumor content, where the extracted DNA originated from a mixture of tumor and non-neoplastic cells, hampering the ability for SNP array to detect abnormalities.

## Current Treatment Options

### Overview

The treatment approach and therapeutic landscape for patients with newly diagnosed and R/R MCL has changed substantially in recent years, largely due to pivotal new therapies and deepened understanding into the molecular alterations in MCL to inform their use. Besides conventional immunochemotherapy (IC), current treatments also include molecularly targeted therapies such as ibrutinib (BTK inhibitor) and venetoclax (BCL-2 inhibitor) and more recently, immune effector cells such as the chimeric antigen receptor (CAR)-modified T-cell product brexucabtagene autoleucel directed against CD19. Of note, clinical trial participation at any stage of treatment is generally encouraged given the rarity of MCL and the pressing need to improve outcomes in this disease.

### Frontline Treatment for MCL

A number of considerations inform the therapeutic recommendations for patients with newly diagnosed MCL who require treatments. Of note, variability exists across treatment centers in this initial treatment approach. In current practice, the greatest patient- and tumor-specific factors of interest are the patient’s age, frailty/comorbidity, and goals for care, as well as factors alluded to in the *Prognostic Factors* section above, namely clinical and biologic features of the disease, including tumor *TP53* alteration status.

Standard frontline treatments include IC or rituximab-lenalidomide (77). Younger, fit patients seeking maximal response duration generally receive multiagent IC followed by autologous stem cell rescue (HDT/ASCR). Conventional IC regimens include R-DHAP (78) (rituximab, dexamethasone, cytarabine, platinum), alternating R-CHOP/R-DHAP (52), and dose-intensified R-CHOP plus cytarabine (79). Maintenance rituximab is typically given post-HDT/ASCR based on data from Le Gouill et al. demonstrating improvements in PFS, event-free survival, and OS (78) with this approach. Of note, given uncertainty regarding the effect of HDT/ASCR on OS, an ongoing randomized study (NCT03267433) is evaluating a MRD-based transplant approach wherein MRD-negative patients following IC are randomized to either rituximab maintenance or HDT/ASCR. Details of MRD molecular testing have been discussed in earlier sections of this review.

Transplant-ineligible patients or those seeking less intensive treatments commonly receive rituximab plus bendamustine (80); in select cases (for example, averse to IC or especially frail or comorbid), patients can also receive rituximab plus the immunomodulatory agent lenalidomide (77).

The clinical importance of *TP53* status is delineated in data from the Nordic Lymphoma Group (64) who evaluated the impact of *TP53* alterations in a cohort of patients who underwent intensive IC followed by HDT/ASCR. They demonstrated poor outcomes in these patients, with a median OS of less than 2 years and a cumulative incidence of relapse of 50% at 1 year. Other groups have demonstrated similar findings (61, 81) to reinforce the prognostic importance of *TP53* alterations. Based on these findings, IC + HDT/ASCR consolidation is not recommended; rather, treatment with novel agents in the context of a clinical trial is favored for upfront treatment of patients with *TP53*-altered MCL.

### Treatment for Relapsed or Refractory MCL

For patients with R/R MCL, the treatment recommendations are highly individualized, and are primarily driven by the extent of disease/symptomatology; time to relapse; response, tolerability, and category/number of prior therapies received; and patient age/frailty/comorbidity and goals for care. Clinically, R/R MCL is characterized by progressive shortening of response duration with subsequent lines of treatment (82) and represents an unmet clinical need. Patients who have experienced favorable PFS with upfront IC may be treated with second-line IC, given the potential for achieving long responses again (83). More commonly, patients are treated with targeted agents. Approved agents (frequently combined with rituximab) include the BTK inhibitors ibrutinib, zanubrutinib, and acalabrutinib (33), the Bcl-2 inhibitor venetoclax (84), and the immunomodulatory agent lenalidomide (**Table 4**) (85). Additionally, many of these agents are being used in combination based on noteworthy preliminary data from ongoing trials [ibrutinib + lenalidomide + rituximab (35) and ibrutinib + venetoclax (37)]. The recent approval of brexucabtagene autoleucel and promising early data for lisocabtagene maraleucel are major landmarks in treating MCL given promising efficacy and tolerability in early results from trials (86, 87). For example, among 32 patients who received lisocabtagene maraleucel (median number prior therapies = 3), the response rate was 84%, including 59% complete response (87). Longer follow-up data are awaited to define the durability of responses with these agents and to inform proper sequencing with other agents in the R/R setting. Future investigations will likely investigate the use of CAR-T cells earlier in the care of patients with MCL (currently only approved for patients with R/R MCL), potentially targeting higher-risk MCL subtypes such as *TP53*-altered or blastoid morphology. Finally, for eligible patients with multiply relapsed MCL, there are supportive data for allogeneic stem cell transplantation, however, the role for this approach along with other therapies will likely evolve given the promise of CAR T-cell therapy.

**Table 4:** Approved targeted therapies for MCL.

Agent	OR rate	CR rate	Median DOR	Median PFS	Citation
Ibrutinib	69.7%	27.0%	21.8 months	12.5 months	(36)
Acalabrutinib	81%	43%	26 months	20 months	(33)
Zanubrutinib	84%	68.6%	19. months	22.1 months	(76)
Venetoclax	53%	18%	8.1 months	3.2 months	(84)
Lenalidomide	40%	5%	16.1 months	8.7 months	(85)

OR, Overall Response; CR, Complete Response; DOR, Duration of Response; PFS, Progression Free Survival.

## Conclusions

Although MCLs are characterized in most cases by t (11,14)(q13q32)/*CCND1-IGH*, other variant translocations and cryptic insertion of enhancer elements can also lead to MCL. There are certain common gene mutations and chromosomal-level alterations for MCLs. Although specific alterations may be more common in either the cMCL or nnMCL subtype, cMCL as a group has higher genomic complexity and higher average number of CNAs on a chromosomal level. As these alterations are accumulated over time during tumor progression, they can affect tumor aggressiveness and prognostication. Many alterations can be detected using widely-available clinical molecular diagnostic tools, including SNP array and targeted/WES assays. On the other hand, studies such as methylation profile assays, are currently mostly performed in the research settings. Nevertheless, detection of relevant genetic alterations is crucial for prognostication and therapy selection. Different frontline and refractory/relapsed treatment options are available for MCL, including immunochemotherapies, targeted therapies, immune effector cell therapy, and stem cell transplantation.

## Author Contributions

SS performed the literature review and wrote the manuscript for this review article. ZE-P and AK researched and wrote the *Current Treatment Options* section. CH researched and wrote the *Important Molecular Diagnostic Tools for Genetic Alterations* section, as well as oversaw the writing of the manuscript in the capacity of the senior author. All authors contributed to the article and approved the submitted version.

## Funding

The authors have been supported by the Comprehensive Cancer Center Core Grant (P30 CA008748) at Memorial Sloan Kettering Cancer Center (MSKCC) from the National Institute of Health, USA (Grant recipient: CT).

## Conflict of Interest

ZE-P received salary support through the AACR-AstraZeneca Lymphoma Research Fellowship and the Lymphoma Research Foundation LSRMP. AK has stocks and other ownership interests in Bridgebio Pharma, consulting or advisory role for Celgene, Kite Pharma (a Gilead company), AstraZeneca, and research funding from: AbbVie/Genetech, Adaptive Biotechnologies, Celgene, Seattle Genetics, AstraZeneca, Pharmacyclics. CH serves on the Hematopathology advisory board for Blueprint Medicines, received honorarium from Invivoscribe, Inc. and Maryland Society of Pathologists, and is a current employee of Loxo Oncology at Lilly.

The remaining author declares that the research was conducted in the absence of any commercial or financial relationships that could be construed as a potential conflict of interest.

## Publisher’s Note

All claims expressed in this article are solely those of the authors and do not necessarily represent those of their affiliated organizations, or those of the publisher, the editors and the reviewers. Any product that may be evaluated in this article, or claim that may be made by its manufacturer, is not guaranteed or endorsed by the publisher.
